# The Many Facets of CD38 in Lymphoma: From Tumor–Microenvironment Cell Interactions to Acquired Resistance to Immunotherapy

**DOI:** 10.3390/cells9040802

**Published:** 2020-03-26

**Authors:** Eleonora Calabretta, Carmelo Carlo-Stella

**Affiliations:** 1 Department of Oncology and Hematology, Humanitas Cancer Center, Humanitas Clinical and Research Center, Rozzano, 20089 Milano, Italy; eleonora.calabretta@cancercenter.humanitas.it; 2 Department of Biomedical Sciences, Humanitas University, Rozzano, 20089 Milano, Italy

**Keywords:** CD38, lymphoma, Daratumumab, immunoescape, checkpoint inhibitors

## Abstract

The CD38 antigen is expressed in several hematological malignancies, and the anti-CD38 monoclonal antibodies Daratumumab and Isatuximab have an established role in the therapy of multiple myeloma. However, data on the therapeutic utility of CD38 targeting in other lymphoid malignancies are limited. In chronic lymphocytic leukemia, the prognostic significance of CD38 expression is well accepted, and preclinical studies on the use of Daratumumab in monotherapy or combination therapy have demonstrated considerable efficacy. In other lymphoproliferative disorders, preclinical and clinical data have not been as compelling; however, CD38 overexpression likely contributes to resistance to checkpoint inhibitors, prompting numerous clinical trials in Hodgkin and non-Hodgkin lymphoma to investigate whether blocking CD38 enhances the efficacy of checkpoint inhibitors. Furthermore, due to its widespread expression in hematological tumors, CD38 represents an attractive target for cellular therapies such as CAR-T cells. The present review discusses current knowledge of CD38 expression and its implications in various lymphoid malignancies. Furthermore, it addresses current and future therapeutic perspectives, with a particular emphasis on the significance of CD38 interaction with immune cells of the tumor microenvironment. Lastly, results of ongoing studies using anti-CD38 antibodies will be reviewed.

## 1. Introduction

The development of the anti-CD38 antibody Daratumumab has redefined the treatment landscape in multiple myeloma (MM), showing impressive anti-tumoral activity in one of the most insidious hematological malignancies [1,2,3,4,5]. Daratumumab, a first in class anti-CD38 antibody, is currently approved both as monotherapy and combination therapy for relapsed/refractory MM (r/r MM) and has shown remarkable activity also in the first-line setting, both for transplant eligible [6] and ineligible [7,8] patients. Currently, Isatuximab, a novel antibody targeting CD38, is in late-stage clinical development, and has shown encouraging responses in r/r MM [9,10,11].

CD38 was first identified in the 1980s in a pioneer study by Reinherz et al., aimed at detecting surface antigens of human lymphocytes using monoclonal antibodies [12], and was initially known as T10. CD38 is predominantly expressed by terminally differentiated plasma cells and their malignant counterpart, but can also be found on the surface of other mature immune cells, such as B cells, T cells, natural killer (NK) cells as well as myeloid cells at early and late stages of development [13]. However, multipotent hematopoietic stem cells lack its expression, suggesting that it is a lineage-defining marker.

CD38 is a multifunctional transmembrane type II glycoprotein, which retains enzymatic activity as well as acting as a receptor. Among its many enzymatic functions, CD38 is involved in the catabolism of intracellular nicotinamide dinucleotide (NAD^+^), in the metabolism of extracellular NAD^+^ precursors and is a major regulator of intracellular calcium homeostasis [14]. In particular, high levels of extracellular adenosine have an increasingly recognized role in cancer biology: it is implicated in promoting immunosuppression via binding to purinergic receptors (the CD38/CD203a/CD73 ectoenzymatic pathway), and may be exploited by T cells of the tumor microenvironment to mediate immune escape. Indeed activation of such pathway correlates with myeloma progression and disease aggressiveness [15,16]. Its receptor component regulates the CD31-mediated adhesion between leukocytes and the endothelial wall, therefore favoring activation and proliferation of leukocytes [13,17,18] and promoting B-cell differentiation.

Biologically, the role of CD38 is less defined, though many hypotheses have been proposed. Firstly, CD38 is thought to have a role in defense against infections: its metabolic functions may limit the availability of NAD^+^ for human pathogens who are obligate NAD^+^ consumers, but lack the ability to synthesize it [19]. Additionally, the accumulation of CD38^+^ inflammatory cells has been associated with aging [20]. Indeed, CD38 modulates the availability of NAD^+^ precursors, which are key players in cell senescence [21]. Finally, it has been suggested that CD38 found in seminal fluid plays a pivotal role in establishing feto-maternal tolerance, though the exact molecular mechanisms remain unknown [22].

Abnormal CD38 expression in hematologic malignancies correlates with cellular proliferation and disease progression, thus making CD38 an attractive target for antibody-based therapeutics. Additionally, its functions in immunomodulation and regulation of intracellular and extracellular metabolic pathways may be targeted to provide indirect anti-tumor activity. Though direct antibody-based targeting of CD38 is well known to produce deep and effective clinical responses in multiple myeloma, data on other lymphoid malignancies are limited.

In this review, we will summarize current knowledge of CD38 expression and its functions in various lymphoproliferative disorders, especially highlighting any therapeutic implications; additionally, we will focus on the emerging role in formation of tumor microenvironment and modulation of immune escape pathways, and, as a consequence, its clinical implications in the era of immunotherapy and cellular therapy.

## 2. Tumor Microenvironment Interactions: Where Does CD38 Stand?

The tumor microenvironment is vital for the development, persistence and progression of cancer, and its possible role as a therapeutic target has been matter of investigation in recent decades in a wide range of malignancies. Immune and non-immune cells cooperate to form a complex network of interactions and alter their activation status to promote tumor survival and dissemination, while evading immune surveillance. In this setting, CD38 expression is commonly found on many immunosuppressive cell types, including (i) regulatory T cells (T regs) (ii) regulatory B cells (B regs), (iii) myeloid-derived suppressor cells (MDSCs) and (iv) the CD16^−^CD56^bright^ NK cell subset, whose importance in tumor—microenvironment interactions is being increasingly recognized.

**Figure 1:**
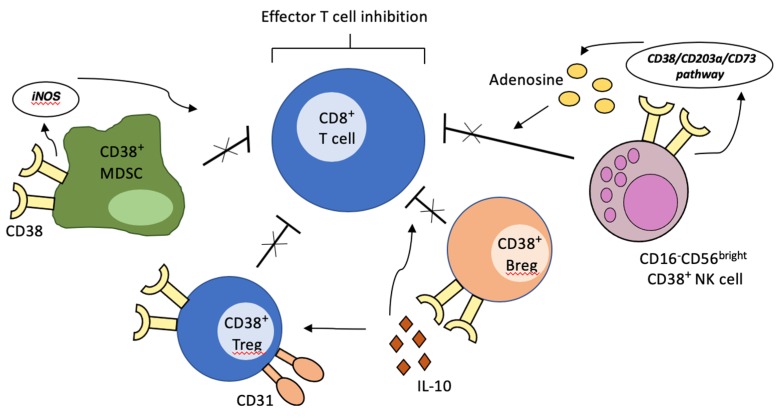
Creation of immunosuppressive microenvironment by CD38-expressing immune cells. The activation of CD38-mediated signaling pathways on regulatory T cells, regulatory B cells, MDSCs and specific NK cell subsets of the tumor microenvironment leads to inhibition of effector T-cell activity, thus contributing to immune escape.

Specifically, conventional CD4^+^Foxp3^+^ regulatory T cells expressing high levels of CD38 have been shown to exert an augmented immunosuppressive activity [23,24]. The underlying mechanism remains unknown. However, CD31^−^ Tregs display a deficient suppressive activity, suggesting that the recognized CD38-CD31 interaction may have a role in this enhanced activity [23]. Accordingly, high CD38 expressing regulatory B cells exhibit a potent immunosuppressive function: by increasing the production of interleukin-10 (IL-10), these cells inhibit T helper 1 and T helper 17 differentiation from naïve T cells, while favoring Treg production [25]. Indeed, deficient activity of Bregs, as identified by low CD38 expression, has been linked, in murine models, to development of autoimmune diseases such as rheumatoid arthritis [25,26]. Additionally, CD38 is a biomarker of MDSCs activity. MDSCs are a recently characterized heterogenous class of myeloid cells with strong immunosuppressive activity, which interact with tumor cells to promote angiogenesis, cancer cell invasion and dissemination [27,28,29,30]. CD38^high^ MDSCs represent an immature form of MDSCs and are characterized by an increased capacity to suppress activated T cells, as a result of increased production of inducible nitric oxide synthase (iNOS). There is evidence that tumor-derived signals directly influence the level of CD38 expression on MDSCs, thus contributing directly to immune escape. Lastly, although classically regarded as cytotoxic anti-tumoral cells, considerable evidence indicates that the CD16^−^CD56^bright^ NK cell subset may be involved in immune regulatory pathways, mainly through the production of anti-inflammatory molecules [31,32], thus depressing effector T-cell proliferation. Morandi and coworkers have recently demonstrated that such inhibitory activity may be related to increased adenosinergic secretion, likely linked to a CD38-dependent pathway [33] (Figure 1).

Collectively, these data suggest that the role of CD38 may go beyond metabolic, enzymatic, and proliferative functions. CD38 may be involved in the formation of a pro-tumoral microenvironment and in its maintenance. Indeed, targeting of CD38 with Daratumumab in MM depletes CD38^+^ MDSCs, Tregs and Bregs and restores cytotoxic T-cell activity [34]. Comparable results have been observed also with the novel anti-CD38 monoclonal antibody Isatuximab/SAR650984 [24].

Additionally, CD38^+^ inhibitory cells have also been identified in other malignancies, including esophageal cancer [35] and colorectal cancer [36], thus expanding their immune regulatory role to solid tumors.

In conclusion, these studies indicate that CD38 is crucial for cell-mediated immuno-modulatory functions and pro-tumoral interactions with components of the microenvironment, thus supporting the use of monoclonal antibodies for the treatment of hematological and non-hematological malignancies. 

## 3. CD38 in Lymphoid Malignancies

### 3.1. Chronic Lymphocytic Leukemia

Chronic lymphocytic leukemia (CLL) is the most common leukemia in adults. CLL is an atypical disease, as the vast majority of leukemic cells in the peripheral blood are in resting state. Proliferation occurs in the so-called “proliferation centers”, located in specific nodal, splenic and marrow niches [37]. The dynamic balance between proliferation stimuli and accumulation of indolent lymphocytes in the periphery determines disease course and aggressiveness, resulting in the highly heterogenous nature of this disease. Identification of markers that may correlate with more aggressive CLL cell subsets is therefore crucial to optimize therapeutical management. In this context, CD38 expression has been regarded as a potential marker of tumor proliferation; its expression fluctuates during the course of the disease and may be acquired or lost by CLL cells according to their proliferation status [38].

CD38 expression was first reported to be associated with an inferior CLL outcome by Damle et al. [39], and later confirmed by subsequent studies [40,41,42], independent of the immunoglobulin heavy chain (IgVH) gene mutational status. CD38 is expressed by approximately 27–46% of patients [42,43]. Levels may increase during the course of the disease, reflecting a switch to a more aggressive phenotype [41,44]; however, CD38 negative patients at diagnosis rarely develop CD38 positivity.

The adverse prognosis associated with CD38 expression is likely related to increased proliferation rate of tumor cells, which become more susceptible to accumulation of pathogenic genetic lesions. Indeed, CD38^+^ cells are present at higher density in the bone marrow and lymph node compartments [45], where interaction with activated T cells and vascular endothelium promotes tumor proliferation [38]. Accumulation in growth-prone lymphoid niches is driven by CD38 expressing neoplastic cells via numerous mechanisms: firstly, CD38 greatly induces the CXCL12-driven chemotaxis of CLL cells by establishing a complex with its receptor, CXCR4 [46]. Additionally, increased homing can be observed as a result of higher integrin-mediated adhesion to VCAM-1: CD38 physically juxtaposes CD49d (i.e., a subunit of integrin α4β1) to Vav-1, which potentiates its phosphorylation and thus the activation of the integrin signaling pathway [47]. Interestingly, high CD49d levels on neoplastic cells strongly correlate with poorer prognosis [48] and development of lymphadenopathy [49], independent of CD38 expression. Lastly, Mele et al., recently demonstrated that CD38 expressing CLL cells exhibit a calcium-mediated Rap1 GTPase activation [50], which is known to have a crucial role in leukocyte trafficking and homing [51], thus elucidating a possible molecular mechanism for the enhanced migration toward proliferation centers.

Investigations into the role of CD38 in CLL and why it correlates with a worse prognosis have provided a more refined insight into the interactions between malignant B cells and microenvironmental components of the “proliferation niche”, as well as uncovering the complex trafficking signals that attract leukemic cells toward the niche itself. Therapeutic implications are promising and will be discussed in detail in the next section.

### 3.2. Mantle Cell Lymphoma

Mantle cell lymphoma (MCL) is a rare type of aggressive non-Hodgkin lymphoma (NHL), comprising approximately 7% of adult NHL in western countries [52]. Due to its aggressive and unpredictable clinical course, most patients are treated with intensive chemotherapy regimens, often including autologous stem cell transplantation. Despite the intensive treatment, virtually all patients ultimately relapse [53]. The approval of the Bruton tyrosine kinase (BTK) inhibitor Ibrutinib for relapsed/refractory patients [54] and the promising activity of the BCL-2 inhibitor Venetoclax [55] have expanded the therapeutic armamentarium. Nonetheless, prognosis remains poor [56].

CD38 expression is frequently found in MCL (approximately 90% of cases) [57] and correlates with nodal involvement and poorer prognosis [58,59]. Additionally, its expression has been implied in resistance to Bortezomib [60]. Collectively, these findings suggest that high CD38 expression promotes clonal B-cell accumulation, as occurs in CLL [61], and may, therefore, represent an attractive therapeutic target.

### 3.3. Follicular Lymphoma

Follicular lymphoma (FL) is the most common indolent NHL. It is characterized by a remitting-relapsing clinical course, with excellent responses to chemoimmunotherapy alternated by phases of disease progression. In addition, approximately one third of patients develop histologic transformation to the more aggressive diffuse large B-cell lymphoma (DLBCL), which is associated with a dismal outcome [62].

Abnormal expression of CD38 is present in approximately two-thirds of FL, though at a lower level compared to more aggressive NHL variants, probably reflecting the lower proliferative burden. Variable levels of expression of CD38 can be detected, however, do not appear to correlate with different histological grades [63].

### 3.4. Diffuse Large B-Cell Lymphoma

DLBCL is the most common histological subtype of NHL, accounting for approximately 30% of all cases [64,65]. Approximately 60% of patients are cured by first-line therapy [66,67]; however, those patients who fail to respond to standard chemo-immunotherapy have a poor prognosis. Efforts have been made to identify predictors of poor response to therapy, and it is becoming increasingly appreciated that DLBCL is an extremely heterogenous disease in terms of clinical features, morphology, immunohistochemistry, and genetic defects. In particular, High-Grade B-cell lymphoma (HGBCL), a new entity of the 2016 revision of the World Health Organization classification of lymphoid neoplasms, is characterized by MYC and BCL2 or/and BCL6 rearrangements, and has a clinical aggressiveness [68], thus representing an unmet medical need.

CD38 is expressed at high levels in most cases of DLBCL, with an average of 80% positivity detected across various tumor specimens [69]. Alsuwaidan et al. have recently identified a highly specific association between the intensity of CD38 expression by flow cytometry (i.e., ‘bright’ or ‘very bright’) and the more aggressive DLBCL variants [70]. Additionally, Sueoka and coworkers have demonstrated that high CD38 expression is an independent predictor of poor outcomes in de novo DLBCL [71]. These findings support the idea that CD38 may reflect the proliferative activity of tumor cells, thus representing a suitable therapeutic target.

### 3.5. Classical Hodgkin Lymphoma

Classical Hodgkin lymphoma (cHL) represents approximately 10% of newly diagnosed lymphomas [72], and is one of the most common malignancies in young adults. It is considered unique among malignancies as it is characterized by a highly immunosuppressive tumor microenvironment comprising the bulk of the tumor, whereas neoplastic cells account for only 1–2%. Within the rich inflammatory infiltrate, Tregs are among the most abundant cell types and actively inhibit the cytotoxic activity of tumor infiltrating lymphocytes (TILs). Di Gaetano et al. have reported that these anergic CD4^+^CD26^−^ T lymphocytes frequently expresses high levels of CD38 [73], thus supporting the idea that CD38 may be a powerful immune-modulatory molecule.

### 3.6. T-Cell Lymphoma

Peripheral T-cell lymphomas (PTCL) account for approximately 8–10% of lymphoid tumors [74] and are generally aggressive, though extremely heterogeneous in terms of clinical presentation, histological subtypes and prognosis. Peripheral T-cell lymphoma, not otherwise specified (PTCL-NOS), and angioimmunoblastic T-cell lymphoma (AITL) are the most frequent subtypes and comprise approximately 30–40% of mature T-cell malignancies [75].

With the exception of the ALK-positive anaplastic large cell lymphoma (ALCL) subtype, nodal PTCLs are generally associated with a poor prognosis, with a 5-year survival of approximately 30%. Standard therapy is based on the administration of CHOP (Cyclophosphamide, Doxorubicin, Vincristine, Prednisone) or CHOP-like regimens and, when feasible, autologous stem cell transplantation; however, options are limited for relapsed disease. There is, therefore, increasing urgency to identify novel therapeutic targets. Recently, Zaja et al. documented that approximately 80% of AITL and 60% of PTCL-NOS express variable levels of CD38, respectively [76], thus providing a rationale for anti-CD38 antibodies in this setting.

Furthermore, CD38 is expressed by the majority of extranodal natural killer/T-cell lymphoma (ENKTL), nasal type, a rare and aggressive subtype of mature T- and natural killer cell lymphomas, commonly associated with Epstein Barr Virus (EBV) infection. Approximately one-half of patients with newly diagnosed ENKTL fail to achieve disease control, and the prognosis in patients with relapsed or refractory disease remains dismal [77]. In this scenario, CD38 has been found to be an independent predictor of poor prognosis [78], and may be therefore targeted by antibody-based therapeutics [79].

## 4. Therapeutic Implications: Daratumumab as Monotherapy or Combination Therapy in Lymphoid Malignancies

In multiple myeloma, targeting CD38 is an established therapeutic approach using the human IgG1 monoclonal antibody Daratumumab. This molecule is known to induce cell death via numerous mechanisms, including complement-dependent cytotoxicity (CDC) [80], antibody-dependent cell-mediated cytotoxicity (ADCC) [80], antibody-dependent cell phagocytosis (ADCP) [81], and apoptosis [82]. Furthermore, Daratumumab effectively targets CD38 expressing myeloid-derived suppressor cells (MDSC) and regulatory T cells, which are known contributors to the formation of an immunosuppressive tumor microenvironment, thus promoting immune escape [34]. In general, Daratumumab is a well-tolerated drug, and its most significant side effects are limited to infusion reactions. Interestingly, it may interfere with disease monitoring by being inappropriately detected as endogenous M protein, although the new test Hydrashift 2/4 has mitigated this effect. Prolonged use of Daratumumab has not been associated with depletion of granulocytes, or significant anemia or thrombocytopenia. Conversely, natural killer (NK) cell counts are decreased, but without consequences in terms of increased susceptibility to viral infections [83]. Lastly, despite inhibiting many immunosuppressive cell types, there is no evidence that it may cause autoimmune phenomena. The relevance of CD38 as a biomarker of clinical aggressiveness in CLL is well known, and its role in tumor homing and proliferation has been elucidated in recent years [84].

Matas-Cespedes et al. have recently demonstrated that treatment with Daratumumab induces effective cell killing in CLL cell lines and mouse models via ADCC and ADCP, whereas CDC activity was negligible [85]. Additionally, Daratumumab inhibits malignant cell homing to the spleen in mice bearing CLL. Finally, it impedes adhesion to VCAM-1 by down-regulating matrix metalloproteinases, therefore inhibiting tumor infiltration and dissemination. Such findings were confirmed by Manna et al. [86], who also detected apoptosis when CLL cells were exposed to Daratumumab. The same research group also detected a CD38-mediated down-regulating of BTK, and demonstrated that accordingly, Daratumumab administration enhances Ibrutinib anti-tumoral activity.

Collectively these preclinical data demonstrate the rationale for investigating the activity of Daratumumab as monotherapy or combination therapy in CLL, especially for those patients who display adverse prognostic factors, such as 17p deletion and/or p53 mutation. Clinical trials evaluating the efficacy of Daratumumab in combination with Ibrutinib are currently recruiting patients (Clinicaltrials.gov).

Evidence of Daratumumab-mediated ADCC and ADCP was also demonstrated in in vitro and in vivo models of NHL (MCL, FL and DLBCL), independently of CD38 expression levels [87]. However, a phase II study assessing the activity of Daratumumab monotherapy in relapsed/refractory NHL failed to produce significant results, with only 8% of patients achieving a response (12.5% of FL and 6.7% of DLBCL) [69]. Although monotherapy with Daratumumab does not exhibit effective cytotoxic activity, combination therapy with standard regimens may enhance response rates. Indeed, Vidal-Crespo et al. have also shown that Daratumumab potentiates the effects of R-CHOP activity in preclinical models of FL, MCL and DLBCL [87]. Clinical studies are thus warranted to assess the benefit of this combination in patients affected by NHL.

Lastly, the report of striking efficacy of Daratumumab in a case of relapsed ENKL [79], has encouraged clinicians to assess its efficacy in a broader patient population. Huang and colleagues have recently disclosed data from a phase II study assessing the efficacy of Daratumumab monotherapy in r/r ENKL: among the 32 patients recruited in the study, the overall response rate was 25%, with a median duration of response of 55 days [88]. Interestingly, response to therapy was not associated with CD38 expression.

Table 1 summarizes currently available results and ongoing phase I/II clinical trials on the efficacy of anti-CD38 antibodies as monotherapy or combination therapy in lymphoid malignancies.

## 5. CD38 in the Era of Immunotherapy and Cellular Therapy

The development of novel pharmacological agents exploiting the activity of the adaptive immune system, i.e., checkpoint inhibitors, and the possibility of directly engineering T cells toward specific tumor antigens, i.e., chimeric antigen receptor T cells (CAR-T cells), has revolutionized the landscape of cancer therapy.

Programmed death 1 (PD-1) is an inhibitory receptor which is expressed, in physiological conditions, by activated T cells. Its ligands, PDL-1 and PDL-2, are present on the surface of antigen-presenting cells. Interaction between these two components results in inhibition of T-cell receptor signaling, which is necessary to blunt the activation of the immune system during inflammation [89]. Recent studies have also revealed that PD-1 signaling preferentially de-phosphorhylates and inhibits the co-stimulatory molecule CD28, which is presently considered to be the prominent mechanism of immune escape in the context of immune checkpoint inhibition [90]. However, many tumors overexpress PDL-1, thus depressing cytotoxic activity of tumor infiltrating T cells, and escaping immune surveillance. PD-1 inhibitors Pembrolizumab and Nivolumab have been developed with the intent of disrupting this ‘exhaustive’ pathway and exploiting the resulting enhanced immune activity for clinical benefit. Indeed, these antibodies have produced unprecedented responses in many malignancies, and have thus been approved for clinical use in advanced solid tumors and for relapsed/refractory (r/r) cHL [91]. Nonetheless, long-term disease control is rare, and most patients ultimately experience disease progression. In this scenario there is compelling urgency to identify potential mechanisms of resistance to checkpoint inhibitors.

**Figure 2:**
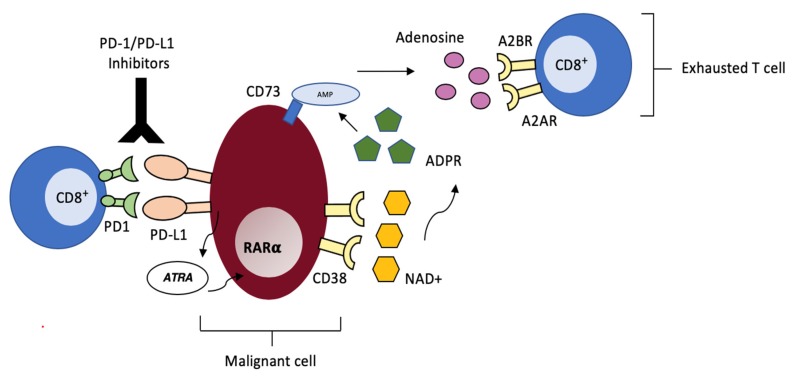
CD38-mediated acquired resistance to PD-1/PD-L1 Inhibitors. Malignant cells resistant to checkpoint inhibition produce mediators such as ATR that lead to CD38 up-regulation via RARα. CD38 catalyzes the conversion of NAD^+^ into immunosuppressive adenosine via the CD38/CD203a/CD73 ectoenzymatic pathway. Adenosine interacts with A2AR and A2BR adenosine receptors on CD8^+^ T cells, thus suppressing cytotoxic activity.

Chen and colleagues have significantly contributed to expanding the knowledge in this field: they have detected up-regulation of CD38 in murine models of lung cancer and melanoma chronically exposed to PD-1/PDL-1 inhibitors, independent of tumor histological type [92]. Up-regulated CD38 on neoplastic cells augments adenosine signaling, which in turn, suppresses CD8^+^ T-cell function, thus providing escape from the adaptive immune system, as had been previously reported by Morandi et al. in melanoma cells lines [93] (Figure 2). Furthermore, they also demonstrated that CD38 knock out tumors exhibit delayed growth when compared to CD38^+^ wild-type tumors in wild-type mice and that in tumor-bearing mice, combination of anti-CD38 antibody and anti-PDL-1 acts synergistically to suppress tumor growth and dissemination. These findings support the use of this combination in the clinical setting in the attempt to overcome resistance to checkpoint blockade, especially in those tumors where PD-1/PDL-1 inhibitors are especially effective, such as r/r cHL or primary mediastinal B-cell lymphoma (PMBCL) [94,95,96,97]. So far, this combination has been investigated in MM, where patient recruitment has been completed and final results are being elaborated (clinicaltrials.gov), and is currently being tested in HL/NHL (Table 1).

T-cell-mediated immunotherapy with a chimeric antigen receptor (CAR) is novel form of adoptive cellular therapy, employing genetically engineered autologous T lymphocytes to target tumor-specific antigens in an HLA-independent fashion. As of 2019, two anti-CD19 CAR-T cell products, Yescarta and Kymriah, have been approved for use in relapsed/refractory NHL including DLBCL, PMBCL and transformed FL [98]. Overall response rates vary between 52% and 83%; however, at least 60% of the patients with aggressive B-cell lymphoma relapse after anti-CD19 CAR-T cell therapy. CD19 antigen loss represents one of the major mechanisms of immune escape, but in many cases CD19 positive relapses have been observed [99,100,101]. Thus, additional investigations are warranted to better understand mechanisms of relapse and identify strategies to overcome such mechanisms.

In this scenario, alternative targets may be useful to overcome escape strategies. As previously described, CD38 is widely expressed on B-cell lymphoma cells and committed precursors, while undetectable on multilineage stem cells, therefore limiting noxious effects on normal lymphogenesis. Thus, Mihara and his team developed an anti-CD38 CAR, and demonstrated that these CD38-specific T cells effectively eliminate B-NHL cells both in vivo and in vitro [102]. Such results were observed also in a subset of B-cell lymphoma cells expressing BMI-1 (B lymphoma Mo-MLV insertion region 1 homolog) and survivin, which are known negative prognostic factors and correlate with resistance to chemotherapy [103]. Furthermore, the same research unit recently demonstrated that anti-CD38-CAR-T cells can abrogate aggressive and chemo-resistant subtypes of DLBCL, such as double hit lymphoma (DHL) and double expressor lymphoma (DEL) cells. This finding is in agreement with the reported high expression of CD38 on aggressive subsets of DLBCL [70]. This therapeutic strategy can be further improved by administering a combination of anti-CD19-CAR-T cells with anti-CD38-CAR-T cells [104].

These findings provide the rationale to expand the use of anti-CD38 CAR-T cells, which is mainly being investigated in MM, to relapsed/refractory NHL. Specifically, anti-CD38 CAR-T cells may represent an effective salvage therapy for those patients who relapse after anti-CD19 CAR-T cells, or may be administered in combination with anti-CD19 CAR-T cells to improve response rates. Currently, this possibility is being investigated in B-cell acute lymphoblastic leukemia lacking CD19 expression or after failure of anti-CD19 CAR-T cells (clinicaltrials.gov).

Lastly, recruiting cytotoxic T cells to malignant cells by means of bispecific antibodies directed toward cancer-associated antigens is a highly promising therapeutic for treating hematologic malignancies. Indeed, Blinatumomab, a bispecific T-cell engager (BiTE) antibody targeting CD3 and CD19, was recently approved for the treatment of relapsed/refractory B-cell acute lymphoblastic leukemia and to achieve minimal residual disease negativity in the first-line setting [105,106]. Anti-CD38/CD3 bispecific antibodies have not yet been clinically developed but have the potential to become curative options for multiple myeloma and other lymphoid malignancies, given the widespread expression of CD38 on malignant cells. Zuch de Zafra and coworkers have recently demonstrated considerable in vitro and in vivo activity of the novel anti-CD38/CD3 bispecific antibody AMG 424 against high and low CD38-expressing cells, such as those previously exposed to Daratumumab [107]. Clinical trials assessing the activity of such therapy in r/r MM are currently recruiting patients (NCT03309111, clinicaltrials.gov), and optimal results may extend their use to other lymphoid malignancies.

## 6. Concluding Remarks

The CD38-NAD^+^ signaling pathway seems to have a relevant role in the formation of a suppressive tumor microenvironment and promotes the activity of inhibitory cell types, such as MDSCs, Tregs, Bregs and certain subtypes of NK cells. Additionally, it appears to be an important driver of resistance to PD-1/PD-L1 checkpoint inhibitors. CD38 cytotoxic antibodies can, therefore, exhibit direct on-tumor activity as well as indirect immunomodulatory anti-tumoral effects. They have been used to treat CD38 positive tumors, specifically MM, with considerable efficacy and a manageable toxicity profile. Substantial preclinical evidence supports their use also in CLL, both as monotherapy or combination therapy with novel agents, such as BTK inhibitors. On the contrary, other lymphoid malignancies appear to be less sensitive to anti-CD38 antibodies, with evidence of modest activity of single-agent Daratumumab in various types of NHL. Nonetheless, Daratumumab and newly developed anti-CD38 antibodies, such as Isatuximab, should not yet be discarded as therapeutic options: indeed, they may find application in combination with standard regimens to enhance cytotoxic response or with checkpoint inhibitors to overcome acquired resistance. Furthermore, anti-CD38 antibodies have yet to be investigated in T-cell lymphomas.

Lastly, CD38 is a widespread antigen which is present on the surface of activated B lymphocytes and may thus be used as the antigenic target for cellular therapies (i.e., CAR-T cells) not only in multiple myeloma but also in non-Hodgkin lymphoma. Combination therapies with anti-CD19 CARs are especially promising.

## Figures and Tables

**Table 1:** Efficacy of anti-CD38 antibodies for the treatment of lymphoproliferative neoplasms, current data, and future perspectives.

Tumor Type	Drug	Patients (*N*)	OS/PFS/ORR	Authors	References
NHL	Daratumumab	36	ORR 8%	Salles et al., Clin Lymphoma Myeloma Leuk 2019	[69]
r/r ENKL	Daratumumab	32	ORR 25%	Huang et al., Blood 2019 [abstract]	[88]
CLL	Daratumumab + Ibrutinib	31 (estimated)	Currently recruiting	-	NCT03447808 (clinicaltrials.gov)
CLL p53 mut	Daratumumab + Ibrutinib	44 (estimated)	Currently recruiting	-	NCT03734198 (clinicaltrials.gov)
cHL, DLBCL, PTCL	Isatuximab + Cemiplimab	130 (estimated)	Currently recruiting	-	NCT03769181 (clinicaltrials.gov)
